# Holothurian Nervous System Diversity Revealed by Neuroanatomical Analysis

**DOI:** 10.1371/journal.pone.0151129

**Published:** 2016-03-17

**Authors:** Carlos A. Díaz-Balzac, María I. Lázaro-Peña, Lionel D. Vázquez-Figueroa, Roberto J. Díaz-Balzac, José E. García-Arrarás

**Affiliations:** 1 Department of Genetics, Albert Einstein College of Medicine, 1300 Morris Park Ave, Ullmann Room 709, Bronx, New York, 10461, United States of America; 2 Department of Biology, University of Puerto Rico–Río Piedras Campus, P.O. Box 23360, University of Puerto Rico, San Juan, PR, 00931–3360, Puerto Rico; Laboratoire de Biologie du Développement de Villefranche-sur-Mer, FRANCE

## Abstract

The Echinodermata comprise an interesting branch in the phylogenetic tree of deuterostomes. Their radial symmetry which is reflected in their nervous system anatomy makes them a target of interest in the study of nervous system evolution. Until recently, the study of the echinoderm nervous system has been hindered by a shortage of neuronal markers. However, in recent years several markers of neuronal and fiber subpopulations have been described. These have been used to identify subpopulations of neurons and fibers, but an integrative study of the anatomical relationship of these subpopulations is wanting. We have now used eight commercial antibodies, together with three antibodies produced by our group to provide a comprehensive and integrated description and new details of the echinoderm neuroanatomy using the holothurian *Holothuria glaberrima* (Selenka, 1867) as our model system. Immunoreactivity of the markers used showed: (1) specific labeling patterns by markers in the radial nerve cords, which suggest the presence of specific nerve tracts in holothurians. (2) Nerves directly innervate most muscle fibers in the longitudinal muscles. (3) Similar to other deuterostomes (mainly vertebrates), their enteric nervous system is composed of a large and diverse repertoire of neurons and fiber phenotypes. Our results provide a first blueprint of the anatomical organization of cells and fibers that form the holothurian neural circuitry, and highlight the fact that the echinoderm nervous system shows unexpected diversity in cell and fiber types and their distribution in both central and peripheral nervous components.

## Introduction

The phylum Echinodermata comprises a distinctive group of marine invertebrates characterized by radial pentameric symmetry originating from bilaterally symmetrical larvae. Five Classes make up this phylum: Crinoidea (sea lilies and feather stars), Asteroidea (starfish), Ophiuroidea (brittle stars and basket stars), Echinoidea (sea urchins and sand dollars) and Holothuroidea (sea cucumbers). Phylogenetically, these organisms are one of the few invertebrate groups within the deuterostome clade, which includes the chordates [1]. The evolutionary position of the Echinodermata makes the study of their nervous system important to understand the evolution of the vertebrate nervous system.

**Fig 1:**
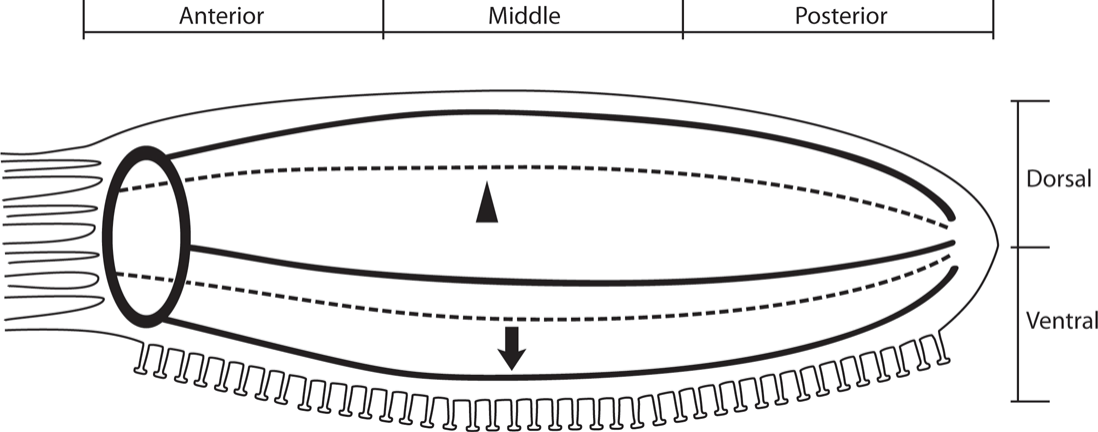
The localization of the holothurian radial nerve cords. The holothurian nervous system is characterized by an anterior nerve ring from which 5 radial nerve cords extend up to the anus. These can be divided into ventral and dorsal according to the localization of the tube feet (ventral). Samples were obtained from the ventro-lateral ambulacrum region (arrow) and dorso-lateral body wall (arrow head), which were divided into anterior, middle and posterior; and from the large intestine.

The major divisions of the echinoderm nervous system have been identified since early last century [2]. Briefly, the main components include five radial nerve cords (RNCs) that connect to a circumoral nerve ring in the oral region of the animal (Fig 1). These RNC and nerve ring comprise the echinoderm central nervous system. In most echinoderms the RNCs are subdivided into the ectoneural and hyponeural subdivisions, separated by a thin layer of connective tissue. In holothurians, the composition of the circumoral nerve ring and the RNCs has been described at the light and electron microscope level revealing the presence and organization of neurons and glia [3, 4]. Peripheral nerves that connect the RNC with other organs, including the viscera, body wall muscles and podia have also been described. Our group has additionally described other peripheral nervous system components in holothurians, including the enteric nervous system, the connective tissue plexus and, more recently, the neural circuitry of the holothurian podia [5–7]. Although these advances serve to elucidate certain aspects of echinoderm neurobiology, they also highlight the scarce information available on the adult nervous system, particularly information on cellular phenotypes, neurochemistry and how these integrate into a working neural circuit. For this reason, most recent studies have focused on identifying markers of the echinoderm nervous system by either using previously available neuronal markers in other organisms or by creating new markers.

Many of these studies were made possible by the development of antibodies such as 1E11 that recognizes echinoderm synaptotagmin [8] or the RN1 antibody, that labels a large fiber and cell components of the nervous system, but whose epitope is still unknown [7]. Smaller subpopulations of neurons have also been characterized by using previously described nervous system markers, particularly neurotransmitters or their synthesizing enzymes or neuropeptides. Thus, the gabaergic and catecholaminergic nervous system components of echinoderms have been described [9, 10] as have been many neurons, neuroendocrine cells and fibers that express neuropeptides. This is the case for SALMFamide [11, 12], GFSKLYFamide [13], galanin [14], and FMRFamide [15]. However, many of these studies have been reported independently of each other, sometimes using different species. Thus, our view of the holothurian nervous system is fragmented and lacks the integration into a working neural circuit of its individual components.

In order to amend this problem we have now used various antibody markers that have previously been reported to identify cells and fibers of the echinoderm nervous system to perform a detailed neuroanatomical analysis of the holothurian nervous system in the species *Holothuria glaberrima*. We present here the labeling pattern of these markers in the RNCs, the longitudinal muscle and the intestine and describe the possible neuronal circuits that can be identified. These findings are of great importance for physiological and developmental studies of echinoderms, as they provide the tools for the identification of nervous system components, as well as outline the physical circuitry that mediates the organism’s behavior. It also sets the basis for the localization of molecules that are emerging from transcriptomic and genomic studies in echinoderms.

## Results

The antibodies used labeled neurons and fibers in the RNC, muscle and intestine of *H*. *glaberrima*. However, the intensity and localization of the labeling varied with each antibody. The labeling by all of these markers has been previously described in *H*. *glaberrima* or other echinoderms [6, 7, 10, 13, 14, 16, 17] and our results closely resembled what has been reported. In the following sections we will describe the presence and localization of cells and fiber in the RNCs, the muscle component and the intestine. For the purpose of making comparisons among the various markers we used serial sections of the ventro-lateral ambulacrum area of the animals, which included the radial nerve cord, the longitudinal and circular muscles, the tube feet, and the large intestine (Fig 1).

### Radial nerves and peripheral nerves

**Fig 2:**
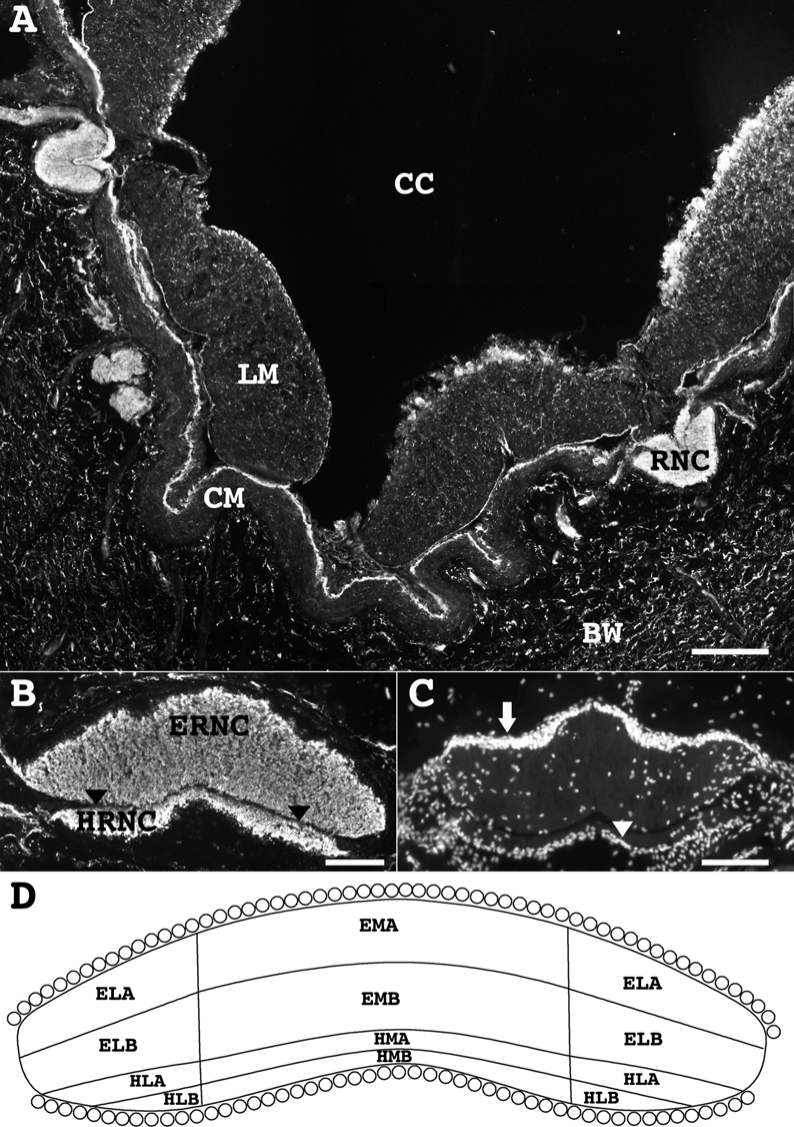
The holothurian nervous system as seen from a transverse section. (**A**) Reconstruction of a transverse section of the nervous system of *H*. *glaberrima* in which the ventral region radial nerves (RNC) and the nervous components of the body wall (BW), longitudinal muscle (LM), and circular muscle (CM), can be appreciated by their immunoreactivity to the RN1 antibody. (**B**) The ventro-lateral ambulacrum region radial nerve is divided into the ectoneural component (ERNC) and the hyponeural component (HRNC) by a thin connective tissue layer (arrowheads), as shown by immunolabeling with the RN1 antibody. (**C**) Neuronal and glia somata in the ectoneural (arrow) and hyponeural (arrowhead) components as observed by DAPI staining. (**D**) For the purpose of this study, each component of the radial nerve was subdivided into central and lateral regions and these regions further subdivided into apical and basal. Scale bar = 300 μm in A; 80 μm in B and C. CC, coelomic cavity; ELA, ectoneural lateral apical region; ELB, ectoneural lateral basal region; EMA, ectoneural medial apical region; EMB, ectoneural medial basal region; HLA, hyponeural lateral apical region; HLB, hyponeural lateral basal region; HMA, hyponeural medial apical region; HMB, hyponeural medial basal region.

RN1 antibody immunostaining in sections of the body wall identified most of the neural structures and showed the extent of the holothurian nervous system (Fig 2). The RNCs are the most prominent neural structures and the principal component of the holothurian nervous system (Fig 2A and 2B). Each of the five RNCs is composed of a central neuropile with some scattered cell bodies and most cell bodies organized in the periphery (Fig 2C). The RNCs are subdivided into the ectoneural (ERNC) and hyponeural (HRNC) regions by a connective tissue structure (Fig 2B). From each component, peripheral nerves extend to innervate their respective target. Most of the peripheral nerves from the ERNC extend to the body wall and innervate the body wall structures, including the dermis, epidermis, and the tube feet. On the other hand, most of the peripheral nerves originating from the HRNC extend to the muscle components and thus innervate the circular muscle (CM) and longitudinal muscle (LM). Some of the markers used in this study identified subpopulations of cells and fibers, or specific neural structures. In order to further describe the various fiber populations, a grid was applied to each RNC and each component (hyponeural and ectoneural) was defined according to its position as lateral-medial, and apical-basal regions (Fig 2D).

#### Fibers

**Fig 3:**
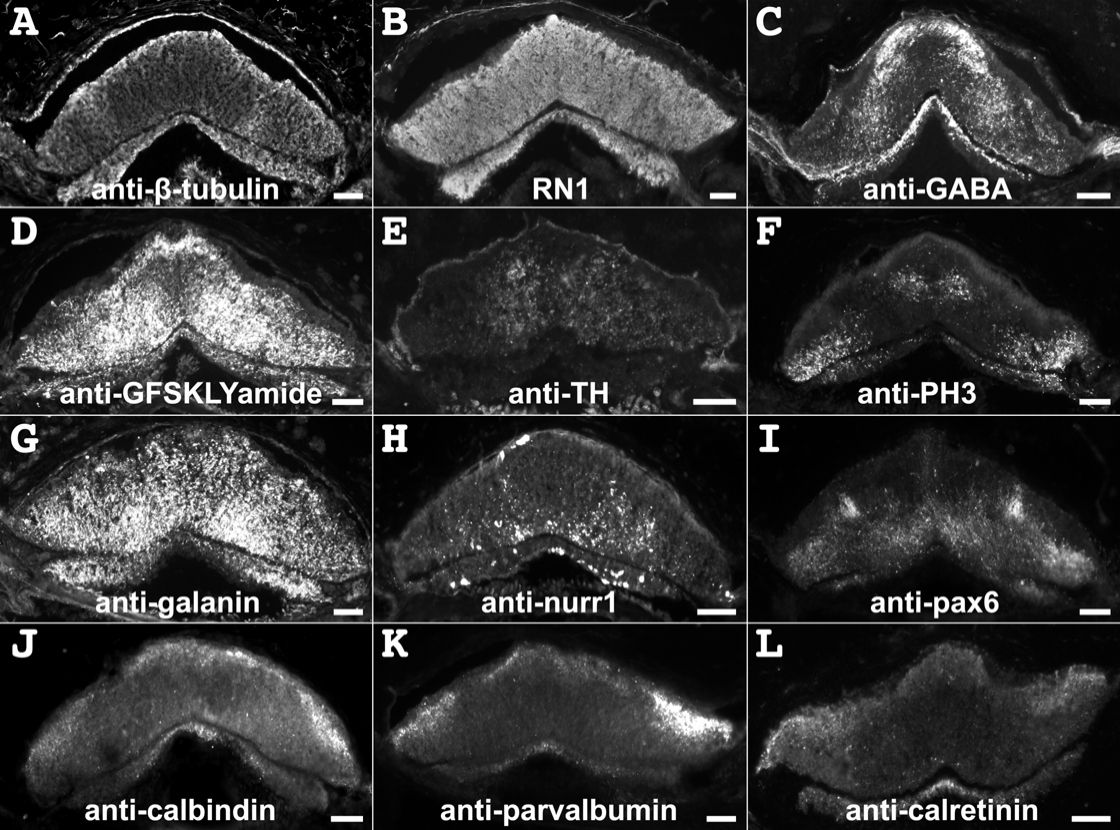
Labeling by neuronal markers of transverse sections of the radial nerve cord is specific to each marker. (**A**, **B**) Anti-β-tubulin and the RN1 antibody labeled extensively both components of the radial nerve cord. (**C**) Anti-GABA immunoreactivity was preferentially distributed to the medial apical region of the ectoneural radial nerve cord (ERNC) and the central region of the hyponeural radial nerve cord (HRNC). (**D**) Anti-GFSKLYFamide immunolabeling was distributed throughout the RNC, except some central and lateral areas of the ectoneural component. (**E**) Anti-TH immunoreactivity was concentrated to the central region of the ERNC, and none was observed in the HRNC. (**F**) Anti-PH3 immunoreactivity was observed in the central and lateral basal regions of the ERNC, and in the lateral regions of the HRNC. (**G**) Anti-galanin immunoreactivity was uniform on all the ERNC, but not much was observed in the HRNC, except on the lateral areas where the hyponeural peripheral nerves form. (**H**) Anti-nurr1 immunoreactivity was observed within the basal region of the ERNC and uniformly through the HRNC. (**I**) Anti-pax6 labeling was very strong in two apical areas of the ERNC, but labeling was also observed in the basal region of the ERNC and uniformly on the HRNC. (**J-L**) Anti-calbindin, anti-parvalbumin, and anti-calretinin immunoreactivity on the radial nerve was very similar, being present in the apical region of the ERNC and in the medial region of the HRNC. Scale bar = 40 μm in A-L.

The labeling of the RNC neuropile can be divided into two different patterns. First, there were markers that appeared to label extensively most, if not all, fibers within the radial nerve cord. Anti-β-tubulin and RN1 antibody labeling fall within this group (Fig 3A and 3B). In contrast, most other markers could be preferentially localized to particular component or specific subpopulations of fibers within the RNC. Immunoreactivity to anti-GABA was observed in both components of the radial nerve, though it was more prevalent in the ectoneural component (Fig 3C). Anti-GABA immunoreactive fibers were mostly distributed to the medial apical section of the ERNC, while in the hyponeural component the immunoreactivity was limited to its apical portion. Anti-GFSKLYFamide immunoreactivity, as reported previously by our group, was widely distributed in both ecto and hyponeural components. However, using the grid we noticed that anti-GFSKLYFamide immunoreactive fibers were not as abundant in the central part of the ectoneural neuropile, as compared to other markers (Fig 3D). More interesting was the finding that in the area where anti-GFSKLYFamide and anti-GABA immunoreactive fibers were scarce was the same area where most of the anti-TH and anti-PH3 immunoreactive fibers were observed. Anti-TH immunoreactive fibers were limited to the central region of the ectoneural component of the RNCs and no immunoreactivity was observed in the hyponeural component (Fig 3E). On the other hand, anti-PH3 immunoreactive fibers were observed to be more predominant in the central region, as well as in the lateral basal regions of the ectoneural component and all throughout the hyponeural component (Fig 3F).

Anti-galanin immunoreactivity was observed equally distributed throughout the ectoneural component, but only a few fibers were identified in the lateral region of the hyponeural component (Fig 3G). Immunoreactivity to the anti-nurr1 and anti-pax6 was observed within both components of the RNCs (Fig 3H and 3I); however, their distribution differed. Anti-nurr1 immunopositive fibers were mostly localized to the hyponeural component of the radial nerve cord and the medial basal region of the ectoneural component (Fig 3H). Fibers immunoreactive to anti-pax6 were distributed throughout the ERNC and the HRNC, but two spots with a large density of fibers were observed in very specific regions of the apical region of the ERNC (Fig 3I). Anti-calbindin, anti-parvalbumin, and anti-calretinin immunoreactive fibers were observed in both components of the radial nerve cord, but a majority of these were localized to the apical medial and lateral portions of the ERNC (Fig 3J–3L). In the HRNC, the anti-calbindin, anti-parvalbumin, and anti-calretinin immunopositive fibers were limited to the medial region (Fig 3J–3L).

**Fig 4:**
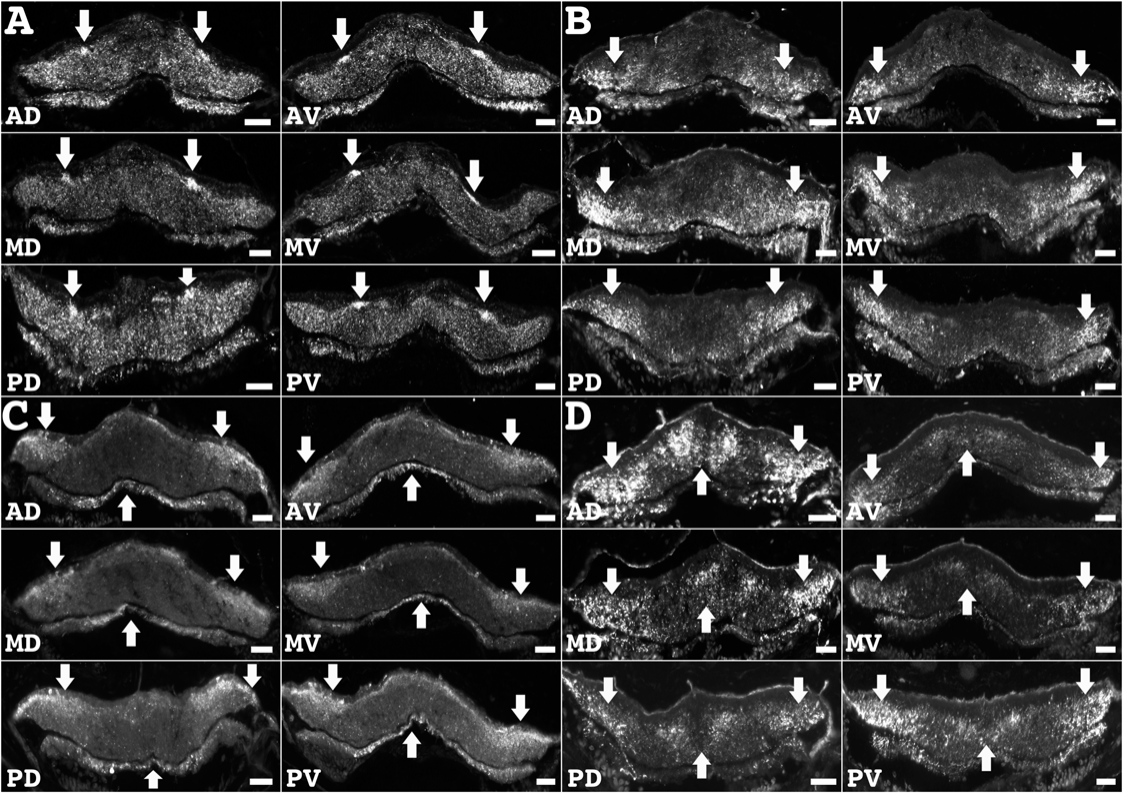
Transverse sections of dorsal and ventral radial nerve cords from different regions of the anterior-posterior axis. (**A-D**) Immunoreactivity to anti-pax6 (**A**), anti-nurr1 (**B**), anti-parvalbumin (**C**), and anti-PH3 (**D**) showing the conservation of the distribution pattern (arrows) of the fibers identified by each marker along the length of the radial nerve cord and comparing dorsal versus ventral radial nerve cords. Scale bar = 40 μm in A-D. AD, anterior dorsal section; AV, anterior ventral section; MD, middle dorsal section; MV, middle ventral section; PD, posterior dorsal section; PV, posterior ventral section.

As explained earlier, these results were obtained from one of the five RNCs that make up the holothurian nervous system and from only one area in the middle axis of the animals. To determine if the immunoreactive patterns observed were present in all RNCs and along the anterior-posterior axis of the animal, we obtained serial sections from the other four RNCs, and from various segments along the anterior-posterior axis of the organism. In general terms, we observed the same pattern of immunoreactivity to be conserved in dorsal and ventral RNCs as well as throughout the length of the RNCs. This was clearly demonstrated by the immunoreactivity of anti-pax6, anti-nurr1, anti-parvalbumin, and anti-PH3 where each one showed their particular fiber pattern though the anterior-posterior axis of the animal, as well as in ventral and dorsally located radial nerves (Fig 4A–4D).

#### Cells

The radial nerves are composed of neuronal and glial cell bodies surrounding the neuropile, although a few cell bodies can be found within the neuropile itself [3, 4]. As shown previously [7], cell bodies were labeled with anti-β-tubulin, but not with the RN1 antibody, while some neurons could be identified with anti-GFSKLYFamide, anti-galanin, anti-TH, anti-GABA, anti-pax6, anti-nurr1, and anti-calbindin [7, 9, 10, 13, 14, 16, 17].

**Fig 5:**
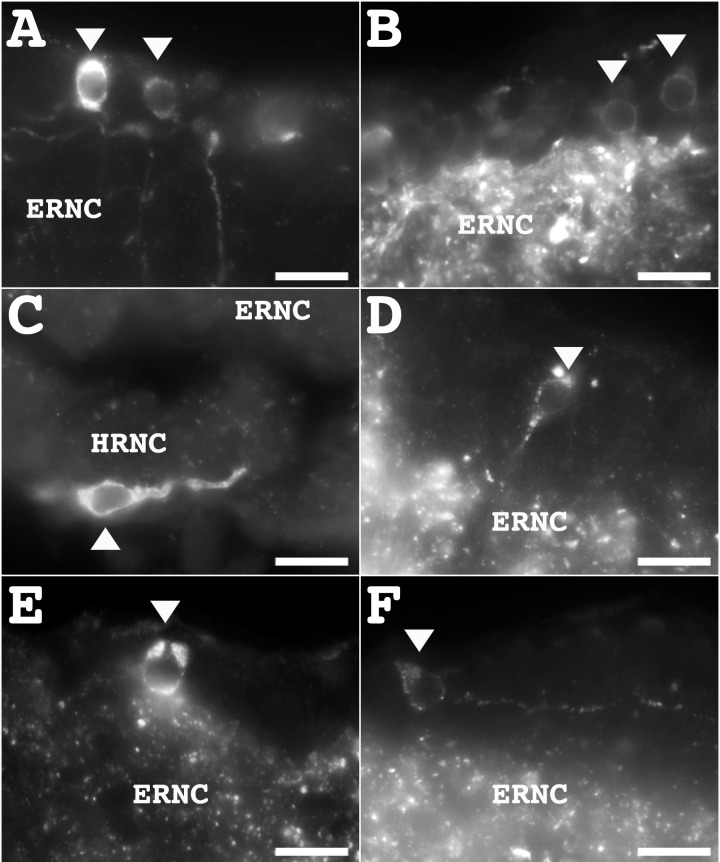
Diversity of neurons identified in the radial nerve cords. Immunoreactive cells were observed in the ectoneural radial nerve cord (ERNC) and/or the hyponeural radial nerve cord (HRNC) as identified by each marker. Catecholaminergic cells were identified by anti-TH in the ERNC (**A**), and cells labeled by anti-calbindin were identified in the ERNC (**B**). Cells labeled by anti-nurr1 were identified in the HRNC (**C**), as well as labeled by anti-GFSKLYFamide (**D**), anti-galanin (**E**), and anti-pax-6 (**F**) were found in the ERNC. Scale bar = 8 μm in A-F.

The markers used in this study identified subpopulations of cells within the RNC, which had particular localizations and morphologies. The first key observation was the large difference in the number of cells labeled by different markers. The biggest cell subpopulation was that identified by anti-TH, corresponding to 23.5% of the cells in the RNC (Fig 5A). These were oval-shaped unipolar or bipolar cells mostly distributed throughout the apical area of the ERNC. The other big subpopulation of cells was that identified by anti-calbindin, corresponding to 20.3% of the cells in the RNC (Fig 5B). These were spherical bipolar cells mostly distributed to the lateral apical areas of the ERNC.

Smaller subpopulations of cells were identified by anti-nurr1, anti-GFSKLYFamide, anti-galanin, anti-pax-6, and anti-GABA. Anti-nurr-1 immunopositive cells corresponded to 3.3% of the cells in the RNC. These were oval-shaped unipolar cells mostly distributed to the medial HRNC (Fig 5C). Anti-GFSKLYFamide immunopositive cells corresponded to 3.5% of the cells in the RNC. These mostly unipolar and oval in shape, and were mostly distributed to the ERNC, but were also observed in the HRNC (Fig 5D). Anti-galanin immunopositive cells corresponded to 0.9% of the cells in the RNC. They were oval-shaped and unipolar and were mostly distributed to the lateral regions of the RNC (Fig 5E). Anti-pax6 immunopositive cells corresponded to 0.4% of the cells in the RNC. These were unipolar and oval in shape, and were mostly distributed to the lateral regions of the RNC (Fig 5F). Anti-GABA immunopositive cells corresponded to 0.5% of the cells in the RNC and were spherical and unipolar mostly distributed to the lateral regions of the ERNC. No gabaergic cells were identified in the HRNC. Finally, anti-PH3 immunopositive cells were rarely observed in the RNC, suggesting that the anti-PH3 somata are found elsewhere. This was indeed confirmed, as we observed anti-PH3 cell bodies in the peripheral nerves associated with the tube feet.

#### Peripheral nerves

**Fig 6:**
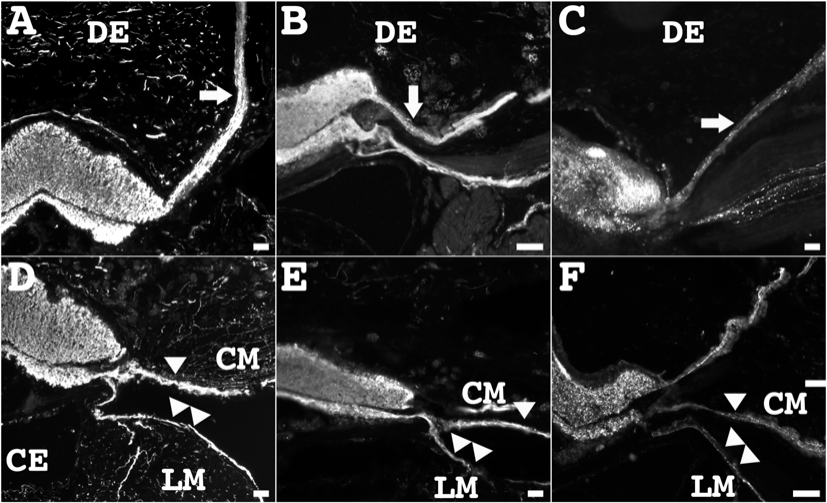
Labeling by neuronal markers of transverse sections of the radial nerve cord identify the peripheral nerves. In a transverse section, the ectoneural peripheral nerve extends from the ectoneural component of the RNC into the dermis (DE) to form the podial nerve (arrow). Immunoreactivity within the ectoneural peripheral nerves was observed with the RN1 antibody (**A**), anti-calbindin (**B**), and anti-pax6 (**C**). In a transverse section, the hyponeural peripheral nerve extends from the hyponeural component of the RNC into the longitudinal muscle (CM) (arrowhead) and longitudinal muscle (LM) (double arrowhead). Immunoreactivity within the hyponeural peripheral nerves was observed with the RN1 antibody (**D**), anti-calbindin (**E**), and anti-pax6 (**F**). Scale bar = 40 μm in C, and E, 150 μm in A, B, D, and F.

**Fig 7:**
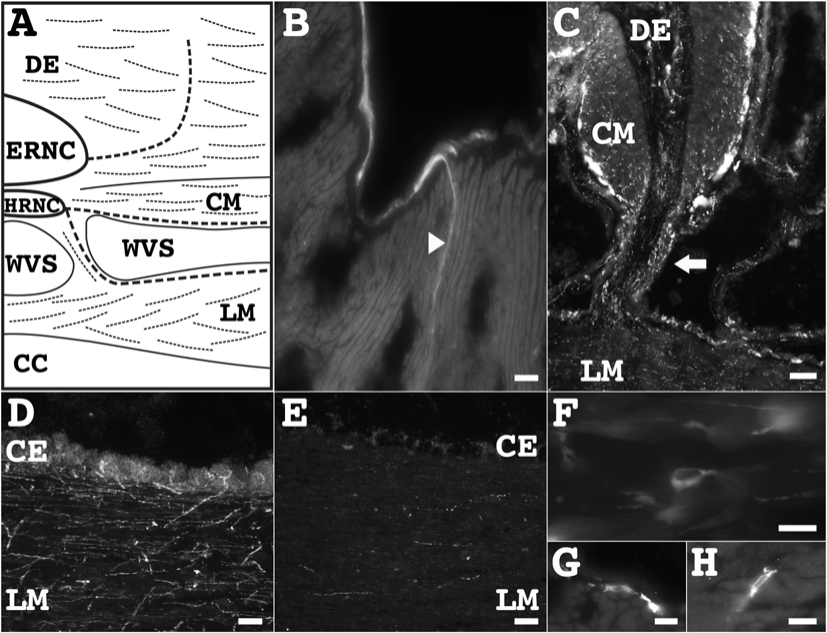
Muscle nervous system components as revealed by immunoreactivity to the RN1 antibody. (**A**) Diagram of a transverse section of the body wall showing the location of the nervous system as identified by immunoreactivity of the RN1 antibody (dashed lines) in relation to the muscle components. The ectoneural peripheral nerve is shown extending from the ERNC to the dermis to form the podial nerve; while the hyponeural peripheral nerve is shown extending from the HRNC to the CM and LM. (**B**) A high magnification view of the LM, showing the hyponeural peripheral nerve within the coelomic epithelia and between the muscle fiber bundles of the LM. (**C**) The RN1 antibody immunoreactivity in fibers and cells of the connective tissue stalk (arrow) that connect the body wall with the LM. (**D, E**) A large subpopulation of fibers in coelomic epithelia and inside the LM was identified by immunoreactivity to RN1 (**D**) while smaller subpopulations were identified by other markers, such as anti-pax6 (**E**). (**F**) Multipolar cells immunoreactive to the RN1 antibody were present inside the LM. (**G, H**) Anti-GABA immunoreactive cells were present in the coelomic epithelia (**G**) and inside the LM (**H**). Scale bar = 40 μm in A, C, D, and E, 20 μm in B, and 10 μm in F-H. CC, coelomic cavity; CM, circular muscle; DE, dermis; ERNC, ectoneural radial nerve cord; HRNC, hyponeural radial nerve cord; LM, longitudinal muscle; WVS, water vascular system.

Peripheral or lateral nerves originate from both RNC components and extend into the periphery. From the ectoneural component of the radial nerve cord, the peripheral nerves extended to the tube feet forming the podial nerve (Figs 6A–6C and 7A). From the hyponeural component of the radial nerve cord, the hyponeural peripheral nerve divided into two nerves, shortly after emerging from the RNC (Figs 6D–6F and 7A). One of the branches innervated the longitudinal muscle while the other innervated the circular muscle. All of the markers showed immunoreactivity within the peripheral nerves extending from both components of the radial nerve cord, but the numbers of fibers identified was different among the markers. RN1 showed the most intensive immunolabeling of the fiber bundle. All other markers labeled minor subpopulations of fibers within the peripheral nerves, and in these cases, individual fibers could be identified. For example, individual fibers expressing anti-calbindin, anti-GABA and anti-GFSKLYFamide immunoreactivities were observed in the peripheral nerves extending from both components of the RNC (Fig 6B and 6E). On the other hand, anti-galanin, anti-PH3, anti-TH, and anti-pax6 immunoreactive fibers were most predominant in the ectoneural peripheral nerve (Fig 6C and 6F), when compared to the fiber population localized within the hyponeural peripheral nerve.

### Motor system

**Fig 8:**
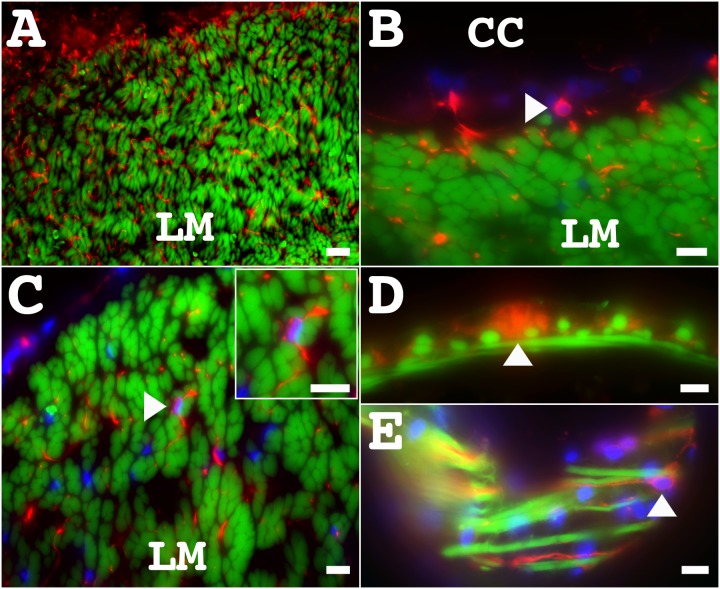
Nervous system components distribution in the muscles and intestinal tissues as revealed by immunoreactivity to the RN1 antibody. (**A**) In a transverse section, many RN1 immunopositive fibers are observed in the epithelia and within the muscle bundles. Higher magnification of the LM shows the presence of RN1 antibody immunopositive cells (arrowhead, insert) and fibers surrounding the muscle fibers. (**B, C**) The RN1 antibody immunopositive cells (arrowhead) localized within the LM coelomic epithelium (**B**) and surrounding the muscle fibers (arrowhead, insert) (**C**). (**D**) RN1 antibody immunopositive fibers bundles (arrowhead) are present in the intestine, associated with the muscle component. (**E**) RN1 antibody immunopositive cells (arrowhead) were also observed to be present in the muscular layer of the intestine. Green = phalloidin labeling of muscle, red = RN1 antibody labeling of neural fibers and cells, blue = DAPI nuclear labeling. Scale bar = 20 μm in A, and 10 μm in B-E. CC, connective tissue cavity; LM, longitudinal muscle.

The two most prominent muscle systems in holothurians are the longitudinal (LM) and circular muscles (CM) of the body wall. In *H*. *glaberrima*, there are five pairs of LMs that run parallel to the anterior/posterior axis. The muscles are adjacent to the RNCs and are attached to the body wall by connective tissue stalks located along the muscle length (Fig 7C). Using the RN1 antibody we were able to define three main subdivisions of the LM innervation. The first subdivision consisted of the peripheral nerve originating from the RNC hyponeural division (Fig 7A). This nerve entered the muscle under the coelomic epithelia and branched out, decreasing in width as it travelled farther into the LM (Figs 6A, 6D and 7A). RN1 antibody labeling also showed fibers and cells localized in the connective tissue stalks that might contribute processes to these peripheral nerves (Fig 7C). The second subdivision was a mesothelial plexus composed of cells and fibers located in the coelomic epithelia that surrounds the LM (Figs 7D, 7E and 8B). The cells localized in this subdivision, were mostly spherical and unipolar and were somewhat abundant, as 10–12 cells could be observed in every tissue section. The fibers within this subdivision appeared to originate from these cells or to extend from the HRNC. The third subdivision consisted of cells and fibers interspersed among the muscular fibers of the LM. In this subdivision, multipolar and oval-shaped cell bodies (6–8 cells per tissue section) were observed (Figs 7F and 8C) as well as two types of fibers. First, there were fiber bundles that corresponded to the branches of the hyponeural peripheral nerve. Second, there were individual fibers, many of which were found closely associated to, or bordering, the muscular fibers. Therefore, when overlays were done with the RN1 antibody and Phalloidin, most of the phalloidin-labeleld muscle fibers were adjacent to RN1 antibody immunopositive fibers. This was also true in other muscles, such as the mesothelia of the intestine, in which the RN1 antibody immunopositive fibers (Fig 8D) and cells (Fig 8E) were observed surrounding most of the muscle fibers.

Except for anti-TH, all markers used in this study labeled fibers within the first subdivision of the LM innervation. In the second and third subdivision, the identification of cells was restricted to a subgroup of the markers, represented by the RN1 antibody, anti-calbindin, and anti-GABA. The morphology and distribution of the cells were similar with the three markers, as that previously described with the RN1 antibody, but the type of cells were not. In both, the second and third subdivisions, anti-GABA immunopositive cells (Fig 7G and 7H) were not co-labeled by the RN1 antibody, while the anti-calbindin immunopositive cells were co-labeled by the RN1 antibody. In summary, there were at least three types of cells and fibers in the second and third subdivisions: (1) GABA^+^, (2) RN1^+^/ calbindin^+^, and (3) RN1^+^.

In addition to the labeling described above there were fibers labeled by anti-nurr1 and anti-galanin but these were restricted to the hyponeural lateral nerve (component 1) and the mesothelium (component 2). Finally, a small number of cells and fibers within the mesothelium (component 2) and interspersed among the muscle fibers (component 3), were labeled by anti-GFSKLYFamide, anti-pax6, or anti-PH3.

### Enteric nervous system

**Fig 9:**
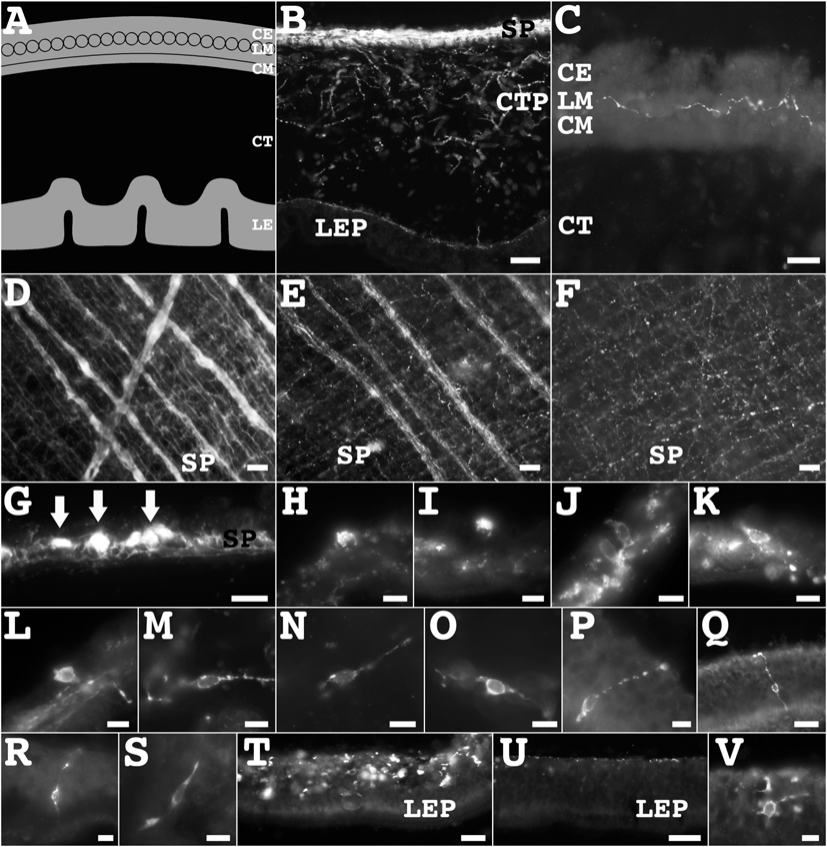
The diversity of the enteric nervous system in *H*. *glaberrima*. (**A**) Diagrammatic transverse section of part of the wall of the large intestine detailing the composition of the main layers of the large intestine. (**B, C**) The main components of the enteric nervous system were identified by most markers, such as the RN1 antibody (**B**); while others, such as anti-PH3 (**C**), identified a small subpopulation of fibers. (**D-F**) Whole mounts of the serosa showing nerves (arrow) and individual fibers within the serosal plexus as shown by their immunoreactivity to the RN1 antibody (**D**), anti-GFSKLYFamide (**E**) and anti-galanin (**F**). (**G**) These nerves correspond to the nerve bundles (arrows) identified in transverse sections of the intestine immunolabeled with the RN1 antibody. (**H-L**) Unipolar and bipolar cells in the serosal plexus were identified by anti-galanin (**H**), anti-GFSKLYFamide (**I**), anti-calbindin (**J**), anti-pax6 (**K**), anti-nurr1 (**L**). (**M-O**) Multipolar and bipolar cells in the connective tissue plexus were identified by the RN1 antibody (**M**), anti-calbindin (**N**), and anti-GABA (**O**). (**P-S**) Bipolar cells in the luminal epithelium plexus were identified by anti-nurr1 (**P**), anti-pax6 (**Q**), anti-GFSKLYFamide (**R**), and anti-calbindin (**S**). (**T, U)** Anti-GABA immunoreactivity identified a novel plexus in the luminal epithelium (**T**), which was not immunoreactive to the RN1 antibody (**U**) or any of the other markers. (**V**) Anti-GABA immunopositive round and unipolar cells were observed in this plexus. Scale bar = 80 μm in B, 40 μm in D-F, 20 μm in C, G-I, S, 8 μm in J-Q, R, T-V. CE, coelomic epithelium; CM, circular muscle; CT, connective tissue; CTP, connective tissue plexus; LE, luminal epithelium; LEP, luminal epithelium plexus; LM, longitudinal muscle; SP, serosal plexus.

The holothurian enteric nervous system (ENS) has been well described by our group [5]. Briefly, it is composed of three main nervous plexus: serosal (also called mesothelial), connective tissue, and the mucosal neuroendocrine plexus (Fig 9A). We now extend the description of the ENS by focusing on the immunoreactivity of some of the new markers that identify particular fiber and cell populations. We also describe two components of the mesothelial plexus, and two distinct components in the mucosa, the gabaergic and the neuroendocrine plexus.

#### Serosal or mesothelial plexus

Our results showed differential expression of markers in the serosal plexus (Fig 9B–9F). Fibers immunopositive to the RN1 antibody, anti-GFSKLYFamide, anti-galanin, and anti-calbindin have been previously reported within the serosal plexus. These fibers can be either found in well-defined nerve bundles or associated with the muscle layer. The nervous markers we used could be divided in three categories (extensive, intermediate and minor labeling) according to the extent that they labeled this fiber plexus. The nerve bundles were easily identified with markers such as the RN1 antibody, anti-β-tubulin, anti-calbindin, anti-GFSKLYFamide, and anti-pax6. The RN1 antibody and anti-β-tubulin, labeled extensively fibers within the nerve bundles, while anti-calbindin, anti-GFSKLYFamide, and anti-pax6 immunoreactivity was intermediate. Although the nerve bundles were not easily identifiable with anti-galanin, anti-GABA or anti-PH3 it is important to note that fibers immunopositive to these markers were observed within the bundles.

All markers labeled fibers associated with the muscle layers in the serosal plexus (Fig 9D–9F). Anti-β-tubulin and the RN1 antibody labeled an extensive number of fibers in the serosal plexus. Anti-calbindin, anti-pax6 and anti-GFSKLYFamide labeled an intermediate number of fibers, co-labeling about 40–50% of the RN1 antibody or anti-β-tubulin labeled fibers. Anti-galanin and anti-nurr1 labeling was intermediate as well, as their immunoreactivity was observed in approximately 20–30% of the RN1 antibody or anti-β-tubulin labeled fibers. Finally, minor subpopulations of fibers were labeled by anti-GABA, anti-TH, and anti-PH3, which corresponded to 1 to 3 fibers, most of which were not co-labeled by the RN1 antibody or anti-β-tubulin.

Although the RN1 antibody and anti-β-tubulin labeled a large number of fibers within the serosal plexus, no cells immunoreactive to these markers were observed (Fig 9G). Cells immunoreactive to anti-galanin (Fig 9H) and anti-GFSKLYFamide (Fig 9I) have been described before and our results confirmed their characteristic immunoreactivity found within granules or vesicles segregated to the two poles of the cells. Additionally, we identified anti-calbindin (Fig 9J), anti-pax6 (Fig 9K), and anti-nurr1 (Fig 9L) immunopositive cells in this plexus. These had a circular morphology and were mostly unipolar, being very different from cells immunopositive to anti-GFSKLYFamide or anti-galanin. Although, the morphology, abundance, and localization of the second group of cells suggested that they correspond to the same subpopulation expressing all the markers, this could not be corroborated by double-labeling because all antibodies were raised in the same species.

#### Connective tissue component or submucosal plexus

In general terms, the presence of nervous fibers within the connective tissue component (CTC) shows a gradient where the area adjacent to the mesothelium contained a large number of fibers, while the density of fibers decreased toward the luminal epithelium. The nervous markers we used could be divided in three categories (extensive, intermediate and minor labeling) according to the extent that they labeled this fiber plexus. Anti-β-tubulin and the RN1 antibody were in the first category as they identified most of the fibers in the CTC. Anti-pax6, anti-GFSKLYFamide, anti-calbindin and anti-galanin were in the second category as they labeled around 25% of the fiber population identified by the RN1 antibody and anti-tubulin, most of these were located close to the mesothelium. Finally, anti-nurr1, anti-PH3, and anti-GABA were in the third category identifying a very small number of fibers (less than 10% of those immunoreactive to the RN1 antibody, which were located close to the mesothelium. No anti-TH immunoreactivity was observed in the CTC plexus.

Cells identified in the connective tissue plexus had a bipolar or multipolar morphology, and were mostly found proximal to the mesothelium. These were immunoreactive to the RN1 antibody (Fig 9M), anti-β-tubulin, anti-calbindin (Fig 9N), anti-GABA (Fig 9O), and anti-nurr1. The RN1 antibody and anti-β-tubulin identified the largest group of cells, while a smaller population of cells was identified by anti-calbindin, anti-nurr1, and anti-GABA. Interestingly, anti-calbindin and anti-nurr1 immunopositive cells were also co-labeled by the RN1 antibody or anti- β-tubulin, while those immunopositive to anti-GABA were not.

#### Luminal epithelium or mucosal plexus

The number of markers that showed immunoreactivity in the luminal epithelium was very limited. In fact, the labeled cells could be divided into two groups according to their morphology and immunoreactivity to a given marker. The first group was identified by their immunoreactivity to anti-nurr1 (Fig 9P), anti-pax6 (Fig 9Q), anti-GFSKLYFamide (Fig 9R), and anti-calbindin (Fig 9S). These cells were mostly bipolar, had an oval shape, and were located in the center of the luminal epithelium. These cells are similar to the anti-GFSKLYFamide labeled neuroendocrine cells. Since all antibodies used to identify the luminal cells were raised in rabbits, we could not perform double-labeling experiments to determine whether the luminal cells represented independent populations or were co-expressing multiple markers.

The second group was that identified by anti-GABA immunoreactivity (Fig 9T). The immunoreactivity labeled a different plexus from that previously described using other markers. These anti-GABA immunopositive cells and fibers, were not co-labeled by neither, the RN1 antibody nor anti-β-tubulin, or observed to be present in serial sections labeled with other markers (Fig 9U). The anti-GABA immunopositive cells were mostly round and unipolar in shape, and were preferentially located proximal to the connective tissue side of the luminal epithelium (Fig 9V).

## Discussion

### The holothurian RNCs are subdivided into nerve tracts

Most of the subdivisions of the echinoderm RNCs were defined by anatomical studies that date to the beginning of last century [2]. The two main subdivisions, the ectoneural and hyponeural components, are easily identified since they are physically separated by a thin partition of connective tissue, but are still inter-connected and constitute a whole anatomical entity [4]. Further experimentation using electron microscopy and/or injury-induced degenerations led to distinctions between putative sensory and motor components [18, 19].

During the past two decades several studies described specific cell and fiber populations in the echinoderm nervous system [5–7, 9, 10, 12–17, 20–22]. However, all of them focus on a specific molecule or epitope, and on the localization of the cells and fibers that express each epitope. What is missing is an integrated study where these markers can be used in the same species to define anatomical subdivisions by the chemical nature of nervous system components, as has been done in many other organisms. Therefore, instead of describing the expression of a single marker we have undertaken the strategy of focusing on three particular anatomical structures and describing the spatial labeling of various nervous markers in the same organism.

It must be stated that we have used the antibody labeling specifically as markers for the anatomical description of specific neuronal and/or fiber populations. Our results should not be interpreted to imply that the molecule recognized by the antibody in the sea cucumber is the same that has been showed to be recognized in other species. Although in some cases the antigen has been described (for example the neuropeptide GFSKLYFamide [13], in other cases the immunolabeling is most likely due to antigen cross-reactivity [16].

The results obtained in *H*. *glaberrima* RNC with the different markers showed that the immunoreactivity to some of these was segregated to specific areas of the RNC. This observation suggests that the radial nerve ectoneural and hyponeural components can be further subdivided according to the cell/fiber neurochemistry. Although most markers identified fibers in both the ectoneural and hyponeural components, albeit with large variability, there is a clear distinction between these two components with the TH marker that only labels cells and fibers in the ectoneural component. Moreover, most of the anti-TH labeling is within the central region of the ectoneural component and no anti-TH labeled cells are observed outside the main neural structures, namely the RNC or peripheral nerves (although some fibers are present in the oesophagus), thus strengthening previous propositions that the TH expressing cells in the RNC are interneurons [10, 23–25].

**Fig 10:**
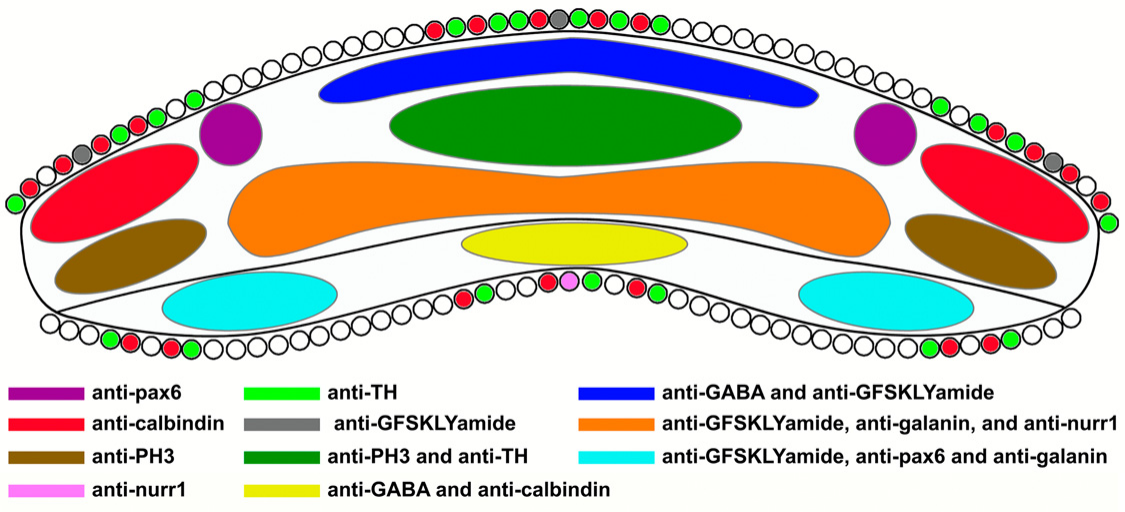
The subdivisions of the radial nerve cord (RNC) in *H*. *glaberrima*. The RNC shows specific areas that can be identified by their specific immunoreactivity to a series of markers. Anti-pax6 (purple) identified two specific regions of large concentrations of immunopositive fibers in the ectoneural radial nerve cord (ERNC). Anti-calbindin (red) identified a subpopulation of fibers in the lateral apical regions of the ERNC, while anti-PH3 (brown) identified a subpopulation of fibers in the lateral basal regions of the ERNC. Anti-GABA and anti-GFSKLYFamide (blue) identified a subpopulation in the medial apical region of the ERNC. Anti-TH and anti-PH3 (dark green) identified a subpopulation in the central region of the ERNC. Anti-GFSKLYFamide, anti-galanin, and anti-nurr1 (orange) identified a subpopulation in the medial basal region of the ERNC. In the hyponeural radial nerve cord (HRNC), anti-GFSKLYFamide, anti-pax6, and anti-galanin (light blue) identified a subpopulation in the lateral region, while anti-GABA and anti-calbindin (yellow) identified a subpopulation in the apical region. Anti-TH (light green) and anti-calbindin (red) identified an extensive group of the cells in the RNC. Most of the anti-GFSKLYFamide (gray) immunoreactive cells are localized to the lateral apical region of the ERNC, while most of the anti-nurr1 (pink) immunoreactive cells are localized in the medial basal region of the HRNC.

The most distinct divisions are summarized on Fig 10. One of the most interesting distributions was that of gabaergic fibers in the medial apical region of the ectoneural and hyponeural components of the RNC. In the asteroid *Asterias rubens* (Linnaeus, 1758) (Asteriidae, Asteroidea), gabaergic fibers have been postulated to be efferent fibers having excitatory or inhibitory effects depending on the effector organ [9]. In the case of the gabaergic fibers localized in the ERNC, these are most likely innervating the body wall and tube feet, while those in the HRNC are innervating the CM and LM. This would coincide with previous research showing that GABA acts as an excitatory neurotransmitter in the tube feet [26, 27] and as an inhibitory neurotransmitter in the longitudinal muscle of echinoderms [9, 28, 29].

Of particular interest is the labeling pattern in the lateral regions of the ectoneural and hyponeural components of the RNC by anti-pax6 and anti-PH3. Most of the labeling with these antibodies can be found in the area adjacent to the peripheral nerves and within the peripheral nerves themselves. This is the area where afferent fibers are expected to enter the RNCs. Since most of the cell bodies labeled with anti-PH3 and anti-pax6 are found outside of the RNCs, this component might be associated with afferent information that reaches the RNCs from sensory structures, such as the tube feet and body wall. In contrast, the reduced number or absence of fibers immunoreactive to anti-GABA or anti-calbindin in this region also supports these observations, as we propose that the latter markers are identifying motoneurons.

Fig 10 also highlights other examples of complementary labeling in which one antibody labels areas of the RNC that are not labeled by another. Such is the case of anti-nurr1 and anti-galanin, or anti-TH and anti-GABA. Anti-nurr1 immunoreactivity was mostly concentrated to the hyponeural component of the RNC, while anti-galanin immunoreactivity within the same area was very scarce. This agrees with previous anatomical observations that suggest that the ectoneural component innervates most of the body wall, while the hyponeural component innervates the LM and CM [2, 22, 30, 31]. Nonetheless 'specialization in innervation' is not always the case in holothurians, since there are purely ectoneural nerves, which innervate longitudinal muscles and the body wall mesothelium [32]. Additionally, anti-TH and anti-PH3 were the only markers that specifically labeled the middle and central regions of the ERNC, suggesting that the anti-PH3 immunopositive sensory fibers may be interacting with interneuron processes, such as those immunoreactive to anti-TH at this site of the ERNC. This supports our observation of the limited distribution of anti-PH3 immunoreactive fibers in major nerves such as the RNC, podial nerve, and the intestinal visceral plexus. This hypothesis is also supported by the fact that immunoreactivity to markers of putative efferent fibers such as anti-GFSKLYFamide or anti-GABA was reduced or absent in the center of the central region of the ERNC where most of the interneuronal processes are located.

It is tempting to speculate that at least some of the regions that are distinctly labeled by markers correspond to the equivalent of fiber tracts within the echinoderm CNS. The best representation of the presence of tracts within the radial nerve cords was the nervous component labeled by anti-pax6. Immunoreactivity to this antigen was specifically identified in two small circular areas in the apical region and in between the medial and lateral subdivisions of the ERNC. This immunoreactivity was continuous through anterior-posterior RNC axis and was similar whether the RNCs were on the dorsal or ventral side of the organism. The significance of this particular localization remains unknown, but recent data may hint to a very specific function for this tract. PAX6 has been reported to be present in the tube feet of adult echinoderms and these structures were shown to be functioning as photosensory organs [33–35]. Therefore, anti-pax6 may identify an afferent tract within the RNC that is carrying information associated with light detection in the organism.

### Holothurian muscles are directly innervated by particular components of the nervous system

Nerves that extend from the HRNC innervate the echinoderm muscles [2, 22, 30, 31]. If the antibody immunoreactivity patterns in the LM and LM neural components are combined with those of the RNC, we can reach important conclusions on possible tracts in the HRNC. Our results support the presence of two main tracts in the HRNC, which may have different functions. The first contains a large number of fibers that are identified by anti-GABA and anti-calbindin. Fibers immunoreactive to either of the markers were predominantly found in the medial apical region of the HRNC. Interestingly, anti-calbindin labeled many cells in the HRNC, while anti-GABA immunopositive cells in the HRNC were very few. Nonetheless anti-calbindin labeling in the HRNC was clearly less than immunoreactivity to the RN1 antibody. There is strong physiological evidence to propose that the main innervation of the LM is cholinergic [18, 28, 36], as acetylcholine has been shown to cause contraction in the LM of the holothurian *Sclerodactyla briareus* (Aurivillus, 1891) [28]. Therefore, it is possible that a population of neurons labeled by RN1 antibody and by the anti-calbindin (RN1+/Calb+) may be cholinergic fibers within the HRNC and LM, as this is the largest subpopulation of cells and fibers present in the HRNC and LM; though further experimentation to confirm this hypothesis is still needed.

The second tract contains the fibers that run in the lateral regions of the HRNC and is composed by a smaller subpopulation of fibers that are immunoreactive to anti-GFSKLYFamide, anti-pax6, and anti-galanin. Interestingly, these markers identified small subpopulations of fibers when compared with the RN1 antibody or to anti-calbindin. This suggests that this tract contains fibers whose function is to control and modulate the muscles. This has been shown to be true in the case of the SALMFamide neuropeptides, such as GFSKLYFamide in *H*. *glaberrima*, which have a relaxing effect on the LM of various echinoderm families [12, 37]. Similarly, histamine and FMRFamide were identified to be present in a small fraction of cells and their processes associated with the muscle of *Leptosynapta clarki*, and histamine was shown to induce peristaltic feeding behavior, suggesting that these neuropeptides might also work as modulators of muscle function [15]. However, further physiological evidence is needed to corroborate the physiological function of these small subpopulations of neurons.

Our observations suggest that the innervation of the muscle is more complex than previously thought. Several of our markers, such as the RN1 antibody and anti-GABA identified subpopulations of fibers adjacent to or surrounding muscle cells, but the numbers of fibers identified were very different. The RN1 antibody and anti-calbindin immunopositive fibers were extremely abundant while those immunopositive to anti-GABA were few in number, further supporting our hypothesis that anti-calbindin is identifying cholinergic neurons in echinoderms. Furthermore, we identified neurons with a distinct morphology and immunoreactivity in the coelomic epithelia and within the muscle fibers, suggesting that the muscle innervation is not limited to the neurons in the HRNC. The cells within the coelomic epithelia are monopolar and send their projections inside the muscle. Whether these cells are motoneurons or sensory neurons is still unknown. Nonetheless, it is important to highlight that these cells differ in morphology and immunoreactivity to those multipolar cells that are localized inside the muscle.

### Novel details on the holothurian enteric nervous system

Our group has previously described the anatomy of the holothurian enteric nervous system [5]. Here, we not only confirm some of our previous findings but also add new details obtained with the new markers. First, nerve bundles are present within the visceral plexus of the intestine. These nerves are readily identifiable with some of the markers that label large subpopulations of fibers such as anti-β-tubulin, the RN1 antibody, or anti-calbindin. Additionally, small numbers or even individual fibers, as those labeled by anti-TH, anti-GABA or anti-PH3, were found within these nerves, suggesting that these nerves are quite diverse and carry both sensory and motor fiber subpopulations.

Second, we identified at least one more type of cell in the visceral plexus, as demonstrated by their immunoreactivity to anti-pax6 or anti-calbindin close to the muscle layers. These are different in morphology, localization, and quantity to the previously described anti-GFSKLYFamide or anti-galanin immunoreactive cells that are localized in the apical side of the mesothelium [13, 14]. Although a clear role for this new group of cells could not be established, we suggest that these cells are analogous to motoneurons in the vertebrate intestine myenteric plexus. It is worth noting that these cells were morphologically similar to those in the coelomic epithelia of the LM, suggesting that these cells might be serving similar functions.

Other novel findings were the anti-pax6, anti-calbindin, and anti-nurr1 immunoreactive cells observed within the luminal epithelium. These are very similar in morphology and distribution to the anti-GFSKLYFamide immunoreactive cells described previously in the same plexus [13]. The possibility that these markers are identifying the same cells could not be corroborated due to our inability to perform co-localization studies with these markers.

Third, there is a gabaergic plexus localized within the luminal epithelium. Previous studies documented the presence of anti-GABA immunoreactivity in small mucosal perikarya in the stomach of the asteroid *A*. *rubens* [9]. Other studies have documented the absence of motor responses in the cardiac stomach of *A*. *rubens* [38] and the oesophagus of *S*. *droebachiensis* (Miller, 1776) (Strongylocentrotidae, Echinoidea) [29], suggesting that gabaergic cells in the luminal epithelium may have other roles in this specific context such as a secretory role.

## Conclusion

In this work we use new, available markers to study and expand our knowledge on the echinoderm nervous system by focusing on the sea cucumber *Holothuria glaberrima*. Using these markers, we have demonstrated that the holothurian nervous system is more diverse than previously thought [2]. First, the RNC of holothurians has specific nerve tracts that are associated with the expression of specific markers. Second, we identified nerves that directly innervate most muscle fibers in the LM. Finally, we show a higher diversity of neuronal subpopulations in one of the best-understood nervous system components of the echinoderms, the enteric nervous system.

The reported findings, together with cumulative evidence gathered within the last decade, should put to rest the notion that the echinoderm nervous system is a net-like system formed by basi-epithelial neurons [39]. Although this type of plexus is indeed associated with the connective tissue of the body wall and of some organs [7], the nervous system of echinoderms shows structures that are characteristic of complex nervous system characteristic structures such as (as shown here) specific fiber populations arranged in well-defined tract-like structures within the central nervous component and nerves within different fiber populations in the peripheral nervous system component.

Our results provide a first blueprint of the cells and fibers that form the holothurian neural circuitry, and highlight the holothurian nervous system unexpected diversity. This diversity is observed by the specific expression of particular antigens; in some cases known neurotransmitters or neural-associated proteins while in others still undetermined antigen that serve to identify neuronal somas or fibers. There are still many questions unanswered, which deserve special attention, such as: What are the molecules the antibodies are identifying in the holothurians? How are the labeled nervous components interconnected? What are their specific functions? What is the evolutionary relationship between the nervous system of echinoderms and that of other deuterostomes? Answering these questions might now be possible with the knowledge that has been obtained during the last decade. Therefore, the better we understand the neuroanatomy of the echinoderms, the easier it will be to elucidate the function and evolution of their nervous system [40].

## Materials and Methods

### Ethics statement

All experiments were performed in accordance with the NIH and NSF guidelines and under the approval of the University of Puerto Rico. *Holothuria glaberrima* are extremely abundant in coastal areas of Puerto Rico. The species is not endangered or protected. They are invertebrate animals and no specific permissions are required for their collection.

### Animals

Adult specimens (10–15 cm in length) of the holothurian *Holothuria glaberrima* Selenka, 1867, were collected from the rocky shores of the north coast of Puerto Rico and kept in sea water aquaria. Use of *H*. *glaberrima* (invertebrates) conforms to the University of Puerto Rico animal regulations. *Holothuria glaberrima* are extremely abundant in coastal areas of Puerto Rico. The species is not endangered or protected. They are invertebrate animals and no specific permissions are required for their collection.

### Tissue sections

Specimens were anesthetized in 0.2% 1,1,1-trichloro-2-methyl-2-propanol (Sigma, St. Louis, MO) for 10 min and dissected by longitudinal section of the body wall. Samples were obtained from the ventro-lateral ambulacrum region and dorso-lateral body wall, which were divided into anterior, middle and posterior; and from the large intestine. The tissues were dissected and fixed in 4% paraformaldehyde at 4°C for approximately 1 h. Tissues were rinsed 3 times for 15 min with 0.1 M phosphate-buffered saline (PBS), and left in a 30% sucrose solution at 4°C for at least 24 h before proceeding to embed them in Tissue-Tek (Sakura Finetek, Torrance, CA). Cryostat tissue sections of 20 μm were cut and mounted on Poly-L-lysine-coated slides.

### Immunohistochemistry

The indirect immunofluorescence method was followed [6, 7, 41]. In brief, tissues were rinsed for 5 min in 0.1 M PBS, followed by a 1 h incubation in goat serum 1:50 (Invitrogen, Carlsbad, CA) and one rinse of 15 min in 1% Triton X, and two other rinses in 0.1 M PBS. Subsequently, the primary antibodies (Table 1) or FITC-labeled phalloidin (Sigma, St. Louis, MO) at a dilution of 1:2,000, were incubated overnight at room temperature. The primary antibodies used include the RN1 monoclonal antibody [7] raised against a homogenate of the radial nerve of *H*. *glaberrima* and used at a dilution of 1:100,000; the monoclonal antibody anti-β-tubulin, (Sigma T-4026 Lot. 024K4862) clone TUB 2.1 prepared against tubulin from rat brain and used at a 1:500 dilution; the rabbit polyclonal antibody anti-GABA (Sigma A-2052 Lot. 067K4769) prepared using GABA-BSA as the immunogen and used at a 1:200 dilution; the rabbit polyclonal anti-pax6 (Abcam ab5790 Lot. 464388) prepared against the synthetic peptide C-REEKLRNQRRQASNTPSHI, corresponding to amino acids 267–285 of Mouse PAX6 and used in at a 1:100 dilution; the rabbit antiserum anti-GFSKLYFamide No. 23 2i2s (Second injection and second bleeding) [13] prepared against a GFSKLYFa synthetic peptide and used at a 1:1,000 dilution; the rabbit antiserum anti-galanin-1 2i3s (Second injection and third bleeding) [14] prepared against galanin (Calbiochem Corp. San Diego, CA) and used at a 1:1,000 dilution; the monoclonal antibody anti-TH [42] raised against tyrosine hydroxylase of quail, *Coturnix japonica*, and used at a 1:100 dilution; the rabbit polyclonal anti-nurr1 (Santa Cruz Biotechnology sc-990 Lot. K1903) prepared against a peptide mapping at the C-terminus of Nurr1 of rat origin and used at a 1:500 dilution; the rabbit polyclonal anti-PH3 (Upstate Biotechnology 06–570 Lot. 21714 and DAM1416518) prepared against KLH-conjugated peptide ARK[pS]TGGKAPRKQLC, corresponding to amino acids 7–20 of human histone H3 and used at a 1:250 dilution; the rabbit polyclonal anti-calbindin (Abcam ab11426 Lot. 378854) prepared against 28 kDa calbindin-D protein purified from rat kidney and used at a 1:500 dilution; the rabbit polyclonal anti-parvalbumin (Affinity Bioreagents PA1-933 Lot. 762–116) prepared against purified parvalbumin from rat skeletal muscle and used at a 1 μg/ml concentration; the rabbit polyclonal anti-calretinin (Abcam ab702 Lot. 721984) prepared against full length Calretinin and used at a 1/100 dilution. Negative controls were performed in all experiments by incubating the tissue sections with rabbit serum during the incubation period of the primary antibody and following the normal immunostaining protocol.

**Table 1:** Antibodies used in this study.

*Antigen*	*Raised in*	*Immunogen*	*Source*	*Dilution used*
Unknown (RN1)	mouse (monoclonal)	Homogenate of the radial nerve of *H*. *glaberrima*	Dr. García-Arrarás lab [7]	1:100,000
β-tubulin	mouse (monoclonal)	Tubulin from rat brain	Sigma (T-4026)	1:500
GFSKLYFamide	rabbit (polyclonal)	GFSKLYFa synthetic peptide	Dr. García-Arrarás lab [43]	1:1,000
Galanin	rabbit (polyclonal)	Galanin	Dr. García-Arrarás lab [43]	1:1,000
Tyrosine Hydroxylase	mouse (monoclonal)	Tyrosine hydroxylase from quail, *Coturnix japonica*	Dr. Fauquet lab [41]	1:100
Calbindin 1	rabbit (polyclonal)	28 kDa calbindin-D protein purified from rat kidney	Abcam (ab11426)	1:500
Parvalbumin	rabbit (polyclonal)	Parvalbumin from rat skeletal muscle	Affinity Bioreagents (PA1-933)	1 **μ**g/ml
Calbindin 2	rabbit (polyclonal)	Full length Calretinin	Abcam (ab702)	1:100
GABA	rabbit (polyclonal)	GABA-BSA	Sigma (A-2052)	1:200
Pax6	rabbit (polyclonal)	synthetic peptide C-REEKLRNQRRQASNTPSHI	Abcam (ab5790)	1:100
Nurr1	rabbit (polyclonal)	Peptide mapping the C-terminus of rat Nurr1	Santa Cruz Biotechnology (sc-990)	1:500
Histone H3	rabbit (polyclonal)	Amino acids 7–20 of human histone H3	Millipore (06–570)	1:250

The secondary FITC antibodies, Goat anti-mouse (Biosource, Camarillo, CA, #AMI0408 Lot. 3501) and Goat anti-rabbit (Biosource, Camarillo, CA, #ALI0408 Lot. 1502)were used at a 1:50 dilution for double labeling indirect immunohistochemistry. Also, the Cy3 conjugated secondary antibodies, Goat anti-mouse (Jackson ImmunoResearch Laboratories, Inc. West Grove, PA, #115-165-068 Lot. 47814) and Goat anti-rabbit (Jackson ImmunoResearch Laboratories, Inc. West Grove, PA, #111-165-144 Lot. 50694), were used at a 1:2,000 dilution for double labeling indirect immunohistochemistry.

Cell nuclei were stained by either a 10 min rinse in 1 **μ**M Hoechst dye (Sigma, St. Louis, MO) or by adding 2 **μ**M DAPI (Sigma, St. Louis, MO) to the buffered glycerol solution used to mount the slides. In cases where double labeling was performed, the two primary antibodies were applied simultaneously and later the two secondary antibodies were added together [41]. Tissues were examined and photomicrographs taken on a Leitz Laborlux fluorescent microscope with N2, I2/3 and D filters or on a Nikon Eclipse E600 fluorescent microscope with FITC, R/DII and DAPI filters. Cells were counted by counting the cells observed in 1 field of view of 3 different sections of 2 different animals. Images were recorded using the MetaVue software (version 6.0; MDS Analytical Technologies, Toronto, Canada) or the Spot Basic software (version 4.7; Diagnostic Instruments, Sterling Heights, MI), and Image J (version 1.37; NIH, Bethesda, MD). These were cropped, brightness and contrast adjusted, using Adobe Photoshop 7.0 (Adobe Systems, San Jose, CA).
